# Peptide specific monoclonal antibodies of Leptospiral LigA for acute diagnosis of leptospirosis

**DOI:** 10.1038/s41598-017-03658-0

**Published:** 2017-06-12

**Authors:** Murugesan Kanagavel, Santhanam Shanmughapriya, Kayanam Vijaya Lalitha Aishwarya, Karuppiah Ponmurugan, Kasi Murugan, Naif Abdullah Al-Dhabi, Kalimuthusamy Natarajaseenivasan

**Affiliations:** 1 Medical Microbiology Laboratory, Department of Microbiology, Centre of Excellence in Life Sciences, Bharathidasan University, Tiruchirappalli, TN 620024 India; 2 Department of Botany & Microbiology, College of Science, King Saud University, Riyadh, 11451 Saudi Arabia; 3 Center for Translational Medicine, School of Medicine, Temple University, Philadelphia, PA 19140 USA; 4 Department of Biotechnology, Manonmaniam Sundaranar University, Tirunelveli, TN 627012 India

## Abstract

Leptospirosis is underdiagnosed due to low sensitivity, need of specialised equipment, and expensive reagents for serological and molecular diagnosis respectively. Considering the sensitivity, rapidity, inexpensive reagents and collection of clinical samples, the monoclonal antibody based antigen detection method from urine samples has been developed and evaluated. LigA (LK90) based B-cell specific epitopes were predicted and synthesised as peptides for the production of monoclonal antibody. LK90_543_: SNAQKNQGNA (amino acids: 543 to 552), and LK90_1110_: DHHTQSSYTP (amino acids: 1110 to 1119) with VaxiJen score of 1.3719 and 1.2215, respectively were used. Thirty two and 28 urine samples from confirmed and seronegative healthy human subjects, respectively were included for the evaluation of MAb-based dot blot ELISA. The specificity of the evaluated MAbs, P1B1 and P4W2 were found to be in the range of ~93–96%. Moreover, the MAbs did not show cross-reactivity with other bacterial antigens as confirmed by IgG ELISA, further validating its specificity for leptospiral antigens. These findings suggest that the developed MAb based dot blot ELISA is a simple, rapid performed in less than 8 h, inexpensive with a ICER of $8.7/QALY, and affordable in developing countries and area where laboratory facilities are limited.

## Introduction

Leptospirosis is an emerging infectious disease of world-wide distribution caused by pathogenic spirochetes of genus *Leptospira* ^1^. The source of infection is either through direct exposures to infected animals or contact with water or soil contaminated with leptospires^2^. It causes various indistinguishable symptoms from mild flu-like illness to fatal hemorrhagic forms with severe involvement of vital organs such as the liver, lung, and kidney^3^. Early and accurate diagnosis is very important for proper and prompt treatment to prevent severe clinical complications of leptospirosis^4^. The gold standard method for the detection of pathogenic *Leptospira* spp. is culture isolation (CI), microscopic agglutination test (MAT) and other serological methods. But these methods suffer from a high risk of culture contamination, poor sensitivity and less reproducibility^5^. To overcome the limitations in conventional or present available diagnostic formats for leptospirosis, there is a stringent demand for the development of new diagnostic formats to enhance sensitivity and specificity during acute illness.

The leptospiral outer membrane proteins have a pivotal role during pathogenesis and generally facilitate to differentiate between pathogenic and non-pathogenic leptospires. Among these, the immunoglobulin like proteins, LigA and LigB adhesins are surface exposed and *in vivo* expressed proteins that interact with the extra cellular matrix and homeostatic proteins of host systems^6^. The diagnostic and vaccine efficacy of LigA protein and their immunogenic epitopes has been studied greatly^6–8^. Thus studies of proteins increased in abundance *in vivo* not only contribute to understanding the host-pathogen interactions, but also are helpful in design of novel diagnostics and vaccines.

The sensitivity and specificity of the aforementioned serological assays are low when performed during the early stage of infection as the appropriate immune response would not have been provoked by the time of sample collection. In this regard, an antigen detection assay that detect circulating leptospiral antigens might offer an effective solution to this difficulty^9^. Assays for the detection of leptospiral antigens and DNA are still being developed^10^, including conventional and real-time PCR assays. However, the molecular methods are unaffordable due to the need for specialized equipment and expensive reagents that are not available during outbreak situations in the field and for routine diagnosis.

MAb based dot-blot assay (antigen capture) could positively be an inexpensive, rapid, and easy to perform diagnostic assay. With this view in the present study we have computationally identified immunogenic epitopes of LK90 (LigA-90 kDa) that can bind B-cells and provoke immune responses. The predicted peptides were synthesized, used to immunize mice and develop monoclonal antibodies that can be utilized in the development of novel antigen detection immunodiagnostic assays. The MAbs obtained were able to detect the native proteins in several leptospiral serovars as evidenced by Western blotting and IgG ELISA. The dot-blot ELISA developed with these MAbs were found to be highly specific for conclusive diagnosis of acute leptospirosis during outbreak situations.

## Methods

### Bacterial strains and culture conditions

MAT was performed to evaluate serological evidence of leptospiral infection^11^. A panel of 13 reference strains were used which included the following serogroups: Australis (serovar Australis, strain Ballico), Autumnalis (serovar Autumnalis, strain Akiyami A and Bangkinang), Ballum (serovar Ballum, strain Mus 127), Bataviae (serovar Bataviae, strain Swart), Canicola (serovar Canicola, strain Hond Utrecht IV), Icterohaemorrhagiae (serovar Icterohaemorrhagiae, strain RGA), Grippotyphosa (serovar Grippotyphosa, strain Moskva V), Hebdomadis (serovar Hebdomadis, strain Hebdomadis), Javanica (serovar Poi, strain Poi), Pomona (serovar Pomona, strain Pomona), Sejroe (serovar Hardjo, strain Hardjoprajitno), Pyrogenes (serovar Pyrogenes, strain Salinem). The strains were obtained from WHO Reference Centre for Leptospirosis, Indian Council of Medical Research (ICMR), RMRC, Portblair. *Leptospira interrogans* serovar Autumnalis strain N2, an isolate from ailing human was also included in the study. All leptospiral isolates used in the study were maintained by regular sub-culturing in Ellinghausen-McCullough-Johnson-Harris (EMJH) bovine serum albumin-Tween 80 medium (Difco Laboratories, USA) at the Medical Microbiology Laboratory, Bharathidasan University, Tiruchirappalli, India.

A panel of bacterial pathogens other than *Leptospira* including *Escherichia coli, Staphylococcus aureus, Serratia marcescens, Citrobacter freundi* and *Pseudomonas aeruginosa* was included as antigens to study the specificity of the developed MAbs. All the bacterial isolates were maintained in Luria-Bertani (LB) agar by regular sub-culturing.

### Study site, Patients, case definition and ethics

The samples recruited for this study were through our routine hospital based surveillance at Annal Mahatma Gandhi Memorial General Hospital, Tiruchirappalli, Tamilnadu, India. In total 32 urine and serum samples from laboratory confirmed leptospirosis cases (a positive IgM ELISA, isolation of leptospires from the blood, seroconversion or four-fold rise in titre by MAT) were collected during the early phase of illness (between 0 and 10 days; Table S1). The age, and sex wise distribution of the confirmed cases included in the study are given (Table S2). A total of 28 seronegative healthy controls matched with respect to age (±5 years) and sex, 28 patients who were hospitalized with a clinical suspicion of leptospirosis and subsequently diagnosed as having other illness based on laboratory evidence, and 25 patients confirmed for Syphilis (13) and Lyme diseases (12) were also included to study the efficiency of the dot-blot ELISA developed in the present study. Informed written consent was obtained from both cases and controls before sampling, and the study protocol was approved by the Institutional Ethics Committee (IEC) of Bharathidasan University (DM/2007/101/373/ Project No. 2) as well as permitted by the Directorate of Health Services (Ref. No. 5796/ TV 1/07), Tamilnadu. The methods were carried out in accordance with the relevant guidelines and regulations.

Rodents were trapped from the same locality of human cases of Tiruchirapalli district. Trap cages were used and additional captures were conducted by locales^12^. A total of 33 urine and serum samples were obtained from field rats and are also included in the study.

Two groups of BALB/c mice were experimentally infected with different serovars of Autumnalis (Group A; 11 animals each infected separately with *L. interrogans* serovar Autumnalis strains N2, Akiyami, and Bangkinang) or EMJH media (Group B). Seven to ten days after infection, serum and urine were collected aseptically. The Institutional Animal Ethics Committee (IAEC) of Bharathidasan University (BDU/IAEC/25/2013/09.04.2013) approved the study protocols. The methods were carried out in accordance with the relevant guidelines and regulations.

### Sample processing and storage

Venous blood samples were collected in tubes without anticoagulant and transported to laboratory on ice. Serum was separated, frozen in aliquots at −80 °C and used to perform MAT and IgM ELISA with live and heat extracted leptospiral antigens respectively for laboratory confirmation of leptospirosis. Individual urine samples from human, rodents and experimentally infected mice were centrifuged at 12,000 × *g* for 10 min at room temperature; the supernatant was discarded leaving an aliquot of 50 μl, boiled for 30 min and centrifuged at 1,000 × g for 10 minutes. The supernatant obtained was subjected for protein estimation by bicinchoninic acid (BCA) method (Sigma-Aldrich, St. Louis, MO) and desired concentration was dotted individually onto NC strips.

### Microscopic agglutination test

Serum samples from patients and rodents were used for MAT. MAT was performed with the panel of 13 leptospiral serovars. Seven days old culture at a concentration of 1–2 × 10^8^ organisms/ml was used as antigen in MAT. Each serum was tested in doubling dilutions starting from 1:20. MAT titers were reported as the reciprocal of the dilution agglutinating ≥50% of live bacterial antigen and a titre of ≥1:80 was considered positive. India being a tropical country and endemic for leptospirosis, a MAT titer of ≥1 in 80 was considered as a cut-off titre for MAT positivity^14, 15^.

### Mouse adapted challenge strains

Mouse Adapted Challenge Strains (MACS) were selected from their corresponding parent strains (reference laboratory strains), by passaging them in BALB/c mice (cyclophosphamide treated) for ~15 times. The MACS strains were passaged twice *in vitro* and used for preparation of heat extracted antigens^13^.

### Preparation of heat extracted antigens

Leptospiral heat extracted antigen was prepared from 7 days old well-grown leptospiral culture (1–2 × 10^8^/ml) of MACS. The cultures were killed by formalin (final concentration 0.5%, v/v), heated in a boiling water bath for 30 min and centrifuged for 30 min at 10,000 × g. The supernatants were used as heat extracted antigens. Protein concentrations were determined by BCA method (Sigma-Aldrich, St. Louis, MO).

### Prediction of LigA specific B-cell epitopes

The protein sequence of LK90 with accession numbers AAB53736 was retrieved from GenBank and subjected for B-cell epitope prediction using BCPred^15^. B-cell epitopes having BCPred score >0.9 and VaxiJEN score >0.4 were selected for further study.

### Peptide synthesis and purification

The peptides that meet the criteria to be highly immunogenic were synthesized by the solid-phase method using 9-fluorenylmethoxy carbonyl (Fmoc) chemistry with biotin at the N-terminus, linked to the peptide sequence through a spacer sequence of “SGSG” and keyhole limpet hemocyanin (KLH) as a carrier protein conjugated at the C-terminus (Peptide 2.0, Inc., USA). The purity of each peptide was ~95% by analytical RP-HPLC and mass spectrometry.

### Immunization and hybridoma production

Six week old BALB/c mice were injected intraperitoneally with 50 µg of synthetic peptides on days 0, 14, 21 and 28. Freund’s complete adjuvant was used for the first dose of immunization and incomplete for the subsequent doses. Three days after the last booster, titre was checked by IgG ELISA. Splenic lymphocytes obtained from mice with the highest ELISA titre were fused with murine SP2/0-Ag14 myeloma cells (NCCS, Pune) in the presence of PEG-1450 (Sigma-Aldrich, St. Louis, MO). The hybridomas were selected in HAT medium and screened for the production of desired antibodies with an IgG ELISA using homologous antigens. Positive hybridoma cells were cloned using limiting dilution to obtain antibodies from a single cell. Purification of antibodies were achieved by ammonium sulfate precipitation of culture supernatant, followed by affinity chromatography using a Pierce Protein G Cartridges (Thermo Scientific, USA) as per manufacturer’s instructions. The purified antibody was analysed by SDS-PAGE and measured quantitatively by UV absorption. The immunoglobulin subclass was determined using a mouse monoclonal antibody IsoQuick typing kit (Sigma-Aldrich, St. Louis, MO) as per manufacturer’s instructions.

### Antigen specificity of MAbs by ELISA

The specificity of produced monoclonal antibodies was tested by IgG ELISA against recombinant LigA (LK90) protein and heat killed antigens prepared from MACS^13^ of different leptospiral serovars, and heat killed antigens from bacterial pathogens other than *Leptospira*. Two micrograms of each representative proteins per well were coated on a flat-bottom polystyrene microtiter plate at 4 °C overnight, using carbonate coating buffer (pH 9.6), followed by blocking with 4% non-fat dry milk. Purified monoclonal antibodies (1:100) in triplicate were added and incubated for 1 h at 37 °C. Bound IgG was detected using HRP-conjugated rabbit anti-mouse IgG (Sigma-Aldrich, St. Louis, MO) at a dilution of 1:8,000. Plates were developed with *o*–phenylenediamine (Sigma-Aldrich, St. Louis, MO). The reaction was stopped with the addition of 50 µl of 1 N H_2_SO_4_, and the optical density was measured at 490 nm (Bio-Rad, USA).

### Dot Blot ELISA

As we propose the final end product of the developed MAb is its utilization in dot-blot ELISA, the specificity of MAbs was tested against recombinant LigA (LK90) protein, heat killed antigens prepared from MACS^13^ of different leptospiral serovars, and heat killed antigens from freshly isolated bacterial pathogens other than *Leptospira*. To assay the utilization of MAbs for antigen capture, one microgram of pretreated urine samples from patients and rodents were dotted individually onto nitrocellulose (NC) strips. Urine samples from healthy individuals and whole cell lysates of leptopsiral serovars were used as negative and positive controls respectively. The NC strips were air dried and blocked with 5% skim milk in PBS (pH 7.4), incubated at room temperature for 1 h and washed thrice (10 mins each) with 0.01 M PBS (pH 7.4). Then the NC strips were incubated with MAbs (5 µg/ml) for 1 h at room temperature. The NC strips were washed with PBST (pH 7.4) as described above and then incubated with HRP conjugated rabbit anti-mouse IgG (1:8,000) for 1 h at room temperature. After incubation the strips were washed (3 times, 10 mins each) and immersed in chromogenic substrate 4-chloro-1-napthol (Sigma-Aldrich, St. Louis, MO) and 30% H_2_O_2_ solution for 5 min, washed with distilled water and air dried. The colour intensity of dots were analysed by Image J software.

### Data analysis

Data were analyzed using Statistica software package version 6.0 (StatSoft, Tulsa, OK). Cut-off values were defined as the corresponding Mean + 2 SD from the sera of healthy controls. Sensitivity was defined as the percentage of the laboratory-confirmed cases of leptospirosis whose serum samples gave mean ODs greater than the relevant cutoff value. Specificity was calculated as the percentage of the control individuals whose samples gave mean ODs below the relevant cutoff value. The positive and negative predictive values (PPV and NPV, respectively) are the proportions of true positives and true negatives, respectively. The percent agreement between the results of the recombinant protein-based ELISA and the results of the MAT, and the corresponding kappa coefficients, were determined using Epi Info version 6.0 (Centers for Disease Control and Prevention, Atlanta, GA)^13^. Graphs were made using SigmaPlot version 11.0 (Systat Software, Inc.) and GraphPad Prism version 5.0 (GraphPad, San Diego, CA). ed.

## Results

### Prevalence of leptospirosis

Among the 32 cases included in the study, circulating anti-leptospiral antibodies were detected by MAT and titres were reported to be in the range between 1:160 and 1:1280. The most frequently observed circulating serovars were Autumnalis (25.4%), followed by Australis (17.8%) and Canicola (15.3%) (Table S3). Similarly, the most frequently encountered serovars among rodents were Javanica (42.4%), followed by Autumnalis (39.4%) (Table S3).

### Prediction of B-cell epitopes

**Figure 1:**
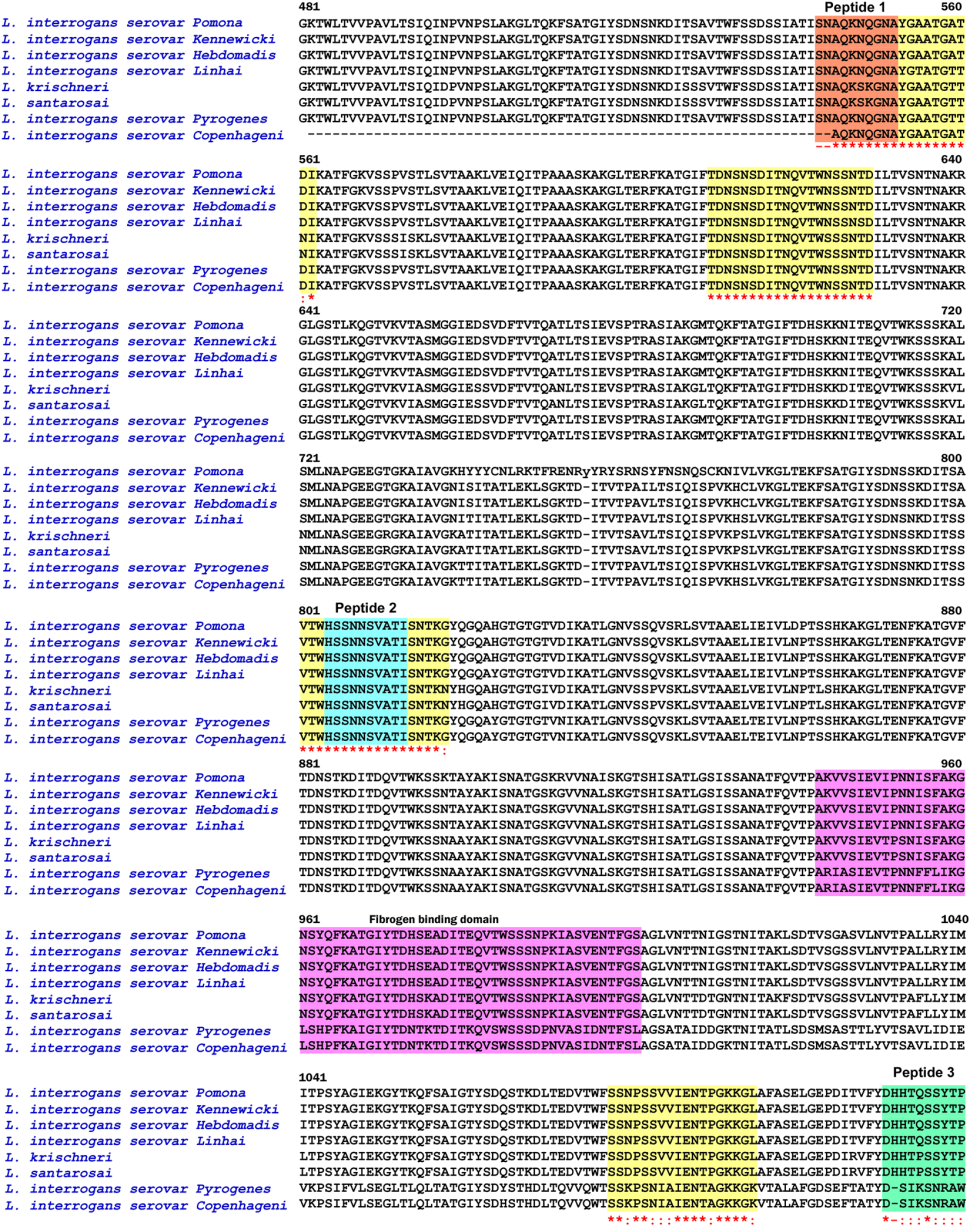
Conservation of the predicted epitopes as determined by multiple sequence alignment among different serovars of *Leptospira*. The LK90 sequences of *L. interrogans* serovar Pomona (GenBank accession number AAN52495), *L. interrogans* serovars Kennewicki (ACH98097), *L. interrogans* serovar Hebdomadis strain R499 (ERK38429), *L. interrogans* serovar Linhai strain 56609 (AJR13073), *L. kirschneri* (WP_020779015), *L. santarosai* (WP_020766576), *L. interrogans* serovar Pyrogenes (EMN28036), *L. interrogans* serovar Copenhageni strain HA10188 (EM018189) were retrieved from GenBank and analyzed with the BioEdit sequence alignment editor (version 7.1.3.0). The predicted B-cell epitopes are highlighted.

The BcPred analysis of LK90 protein sequence predicted eight immunogenic B-cell specific epitopes (Table S4) with VaxiJen score ranging between 0.96 and 0.99. Of the eight predicted epitopes, 2 peptides; LK90_543_: SNAQKNQGNA (aminoacids: 543 to 552), and LK90_1110_: DHHTQSSYTP (aminoacids: 1110 to 1119) with the highest VaxiJen score were selected, synthesized and used for immunization and hybridoma production. The conservation of the epitopes was determined by multiple sequence alignment. The LK90 sequences of *L. interrogans* serovar Pomona (GenBank accession number AAN52495), *L. interrogans* serovar Pomona type kennewicki (ACH98097), *L. interrogans* serovar Hebdomadis strain R499 (ERK38429), *L. interrogans* serovar Linhai strain 56609 (AJR13073), *L. kirschneri* (WP_020779015), *L. santarosai* (WP_020766576), *L. interrogans* serovar Pyrogenes (EMN28036), *L. interrogans* serovar Copenhageni strain HA10188 (EM018189) were retrieved from GenBank and analyzed with the BioEdit sequence alignment editor (version 7.1.3.0). LK90_543_ was found to be highly conserved among different serovars of *L. interrogans* and different species of *Leptospira* and LK90_1110_ was also found to be conserved except for *L. interrogans* serovars Pyrogenes and Copenhageni (Fig. 1).

### Hybridoma production and isotyping of MAbs

A total of 2 × 10^8^ spleen cells obtained from immunized mice with the highest titre in indirect ELISA (1:10240) were fused with 2 × 10^7^ myeloma cells. The culture supernatants from growing hybridomas were screened for antibody production with homologous peptides by IgG ELISA. More than ten hybridomas were found growing in each well of a microtitre plate after fusion yielding approximately 952 and 1243 hybridomas for LK90_543_ and LK90_1110_ respectively. The primary screening identified 62 (6.5%) and 82 (6.6%) hybridomas positive for LK90_543_ and LK90_1110_ respectively. Stable hybridoma clones for LK90_543_ and LK90_1110_ were designated as P1C5, P1B1 and P4E8, P4D1, P4W2 respectively. The produced MAbs had different isotypes, 4 (P1B1, P1C5, P4D1 and P4E8) of IgG1 types and one (P4W2) of IgG2b type and all with kappa light chains.

### Antigen specificity of MAbs

**Figure 2:**
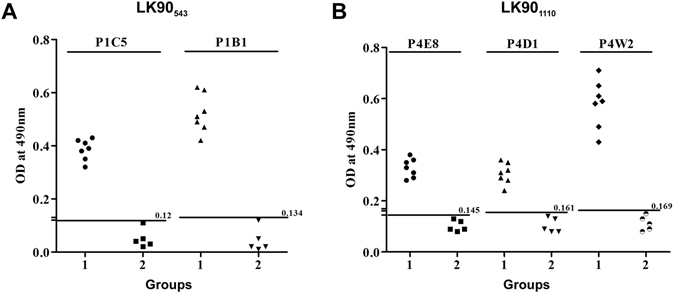
Specificity of the monoclonal antibodies for detection of leptospiral serovars. Heat extracted proteins from MACS leptospiral strains or non-*Leptospira* bacterial pathogens were used as antigens in IgG ELISA. Specificity of the MAbs produced from the hybridoma clones of LK90_543_ (**A**) and LK90_1110_ (**B**) are shown. Study groups: Group 1- Different leptospiral serovars; Group 2- Non-*Leptospira* bacterial pathogens. The line represents the cut-off as determined by Mean + 2 SD of the OD values of other bacterial pathogens, with the absolute values on the right. Study groups are indicated on the x axis and the optical density (OD) at 490 nm on the y axis.

**Figure 3:**
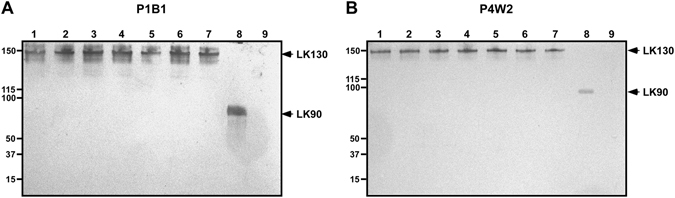
Specificity of the MAbs P1B1 and P4W2 for detection of leptospiral seovars. Heat extracted proteins from MACS leptospiral strains, *E. coli*, and recombinant LK90 were separated on SDS-PAGE and Western blotted with the indicated MAbs. Heat extracted proteins from *L. interrogans* serovars Autumnalis strain Akiyami (Lane 1), Bangkinang (Lane 2) and N2 (Lane 3), serovar Australis (Lane 4), serovar Icterohaemorrhagiae (Lane 5), serovar Pomona (Lane 6), serovar Bataviae (Lane 7), recombinant LK90 (Lane 8) and *E. coli* (Lane 9). P1B1 and P4W2 were found to be highly specific with no cross reactivity to *E*. *coli* extract.

The specificity and reactivity of the MAbs against leptospiral serovars and recombinant LK90 protein were determined by Western blot analysis and ELISA. The overall results of IgG ELISA for specificity and reactivity is given in Fig. 2. The mean + 2 SD of the absorbance values for other bacterial antigens were defined as the cut off value. The cut-off values for P1C5, P1B1, P4E8, P4D1, and P4W2 were 0.12, 0.134, 0.145, 0.161, and 0.169 respectively. The clone P1B1 and P4W2 were found to be highly specific for leptospiral serovars and hence selected for further experiments. To further confirm the specificity of the hybridomas, heat killed antigens of different leptospiral serovars (MACS), *E. coli*, and the recombinant LK90 protein were separated on SDS-PAGE and probed with purified P1B1 and P4W2 MAbs. The produced MAbs did not show cross-reactivity with *E. coli* further confirming its specificity for leptospiral antigens (Fig. 3). The specificity of P1B1 and P4W2 to detect leptospiral antigens were found to be in the range of ~93–96%. In order to further validate the specificity of the MAbs to detect only leptospiral antigens, dot blot ELISA was performed utilizing heat killed leptospiral antigens (MACS), and antigens from other pathogenic bacteria. The dot blot ELISA show the produced MAbs to be highly specific to leptopsiral antigens with no detectable cross reactivity with other antigens (Fig. S1).

### Dot-blot ELISA

**Figure 4:**
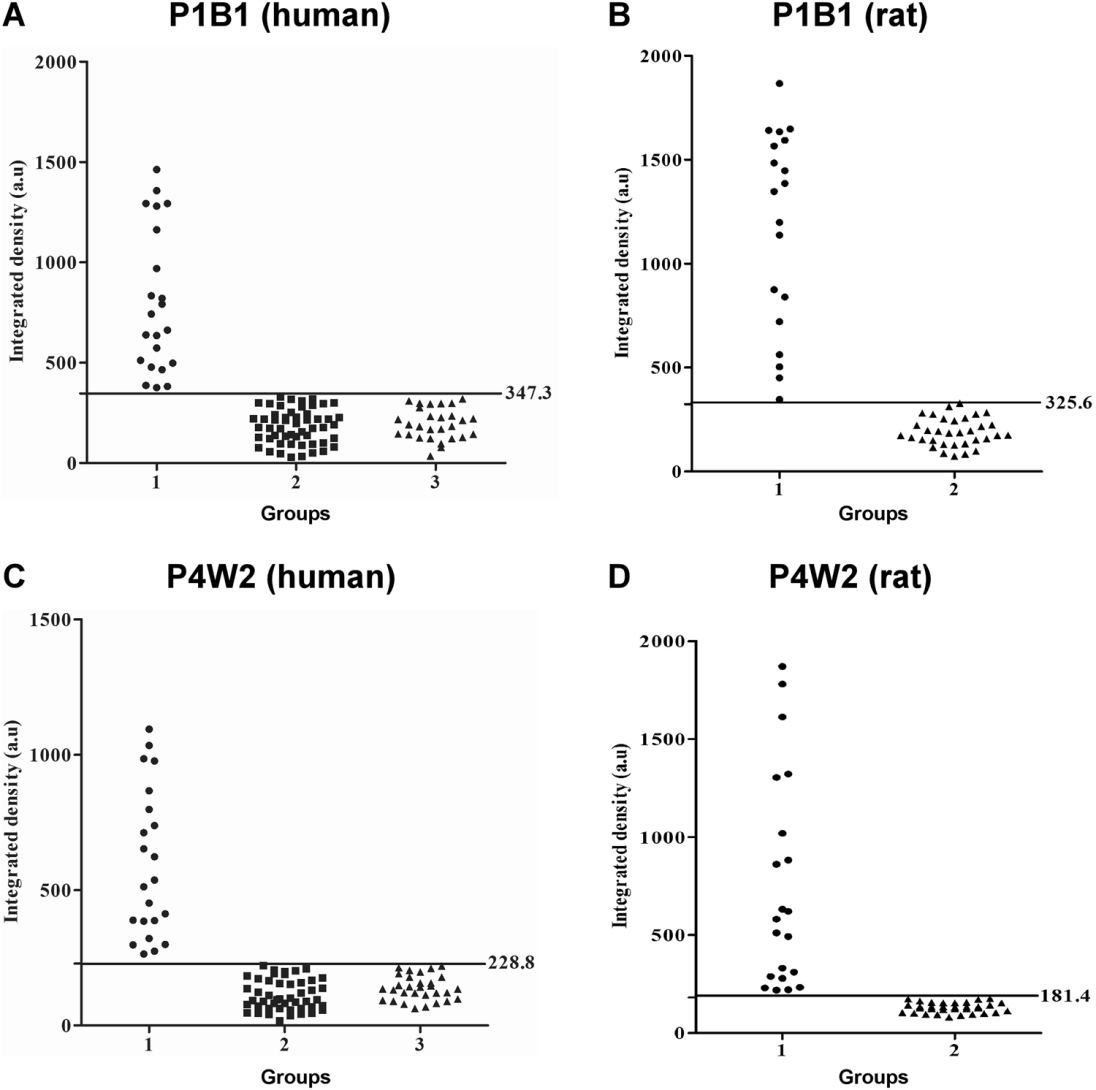
Evaluation of MAbs for antigen-capture in dot blot ELISA for diagnosis of leptospirosis. Study groups are indicated on the *x* axis and intensity of the dots on the *y* axis. Sensitivity and specificity of the MAb based dot blot ELISA for P1B1 (**A,C**) and P4W2 (**B,D**) are shown. Study groups for humans: Group 1 - Laboratory confirmed cases of leptospirosis; Group 2- Confirmed for other infectious diseases; Group 3 - seronegative healthy controls. Study groups for rats, Group 1- seropositive samples, and Group 2 - seronegative rats. The line represents the cut-off with the absolute values on the right.

Dot blot ELISA was developed with urine samples collected from human subjects and rodents. The optimal concentration of antigen that can be captured by MAbs was determined to be 1 μg (Fig. S2). Then we asked whether the developed MAbs can be employed in an antigen-capture dot blot format for diagnosis of leptospirosis. To test this, we first determined the concentration of MAbs that can detect letopsiral antigens (Fig. S3). The optimized concentration of MAbs were determined to be 5 μg and was used in dot blot ELISA. The specificity of the MAbs to capture leptospiral antigen in urine was determined using samples collected from experimentally infected BALB/c mice. The MAbs showed 100% specificity and sensitivity in capturing the leptospiral antigen (Fig. S4). Further we determined the diagnostic efficacy of the MAbs using human and rodent samples. The overall sensitivity and specificity of the Ag-capture dot-blot ELISA developed with MAbs are given in Table 1 and Fig. 4. The specificity of P1B1 and P4W2 to detect leptospiral antigens were found to be in the range of ~93–96%.

**Table 1:** Sensitivities, specificities, positive predictive values (PPV), and negative predictive values (NPV) for dot-blot ELISA using MAbs.

Monoclonal antibody	Urine samples	Sensitivity (%)	Specificity (%)	PPV^a^ (%)	NPV^b^ (%)
P1B1	Human	88.9	96.0	80	98
	Field rat	82.6	93.0	86.4	90.9
P4W2	Human	76.9	95.7	83.3	93.6
	Field rat	84.6	92.5	88.0	90.2

^a^PPV, positive predictive value.

^b^NPV, negative predictive value.

### Calculation of Incremental-cost effectiveness ratio (ICER)

After establishing the importance of dot-blot ELISA in the early diagnosis of leptospirosis, we intended to evaluate the cost effectiveness of the developed strategy. We used decision tree model to determine the cos-effectiveness. The decision tree was made based on the sensitivity of the dot-blot ELISA. Incremental cost effectiveness ratio (ICER) was calculated on comparison with the gold standard MAT. The parameters considered were the probability of each consequence (sensitivity; true positive), calculated approximate cost of the consequence, and the Quality Adjusted life years (QALY). Note: most febrile illness and particularly leptospirosis progress over a 10–15-day period, during which the patients make various possible transitions. Hence a 15-day limit was applied because most patients became afebrile within this period. Thus the model estimated the QALY as the number of *no-fever days* associated with each strategy. Our primary comparison was between; Dot-blot ELISA: sensitivity of 89%, approximate cost of $2.2 per test, and diagnostic delay of 4 days; MAT: sensitivity of 50%, cost of $4.5 per test and approximately diagnostic delay of 10 days. The model yielded an Incremental Cost-Effectiveness Ratio (ICER) as $8.71/QALY (Fig. S5; and Table S5).

## Discussion

Leptospirosis is a serious public health problem that is generally underdiagnosed. The misdiagnosis of the disease is highly fatal due to potential damage to multiple organs. The method most commonly used for the diagnosis of human and animal leptospirosis is detection of specific serum antibody by MAT, and ELISA. These serological methods depend on the presence of circulating antibody and have variable sensitivities and specificities. And moreover, antibodies are detected in the blood approximately 4–7 days after the onset of the symptoms. The development of an early, sensitive and reliable diagnostic format would improve quality of patients’ life. A large array of advanced diagnostic formats including qPCR to detect circulating antigens in blood have been developed^16^. However these diagnostic techniques suffer from several drawbacks like, available only in well-equipped laboratories, needs specially trained persons for performing the assay and not all clinical laboratories achieve same level of diagnostic excellence. Therefore, there is an increased search for a simpler and rapid technique that can be performed in any diagnostic laboratories with minimum laboratory facilities including a table top centrifuge to prepare antigenic samples, and a refrigerator to store specimens. In the acute phase of illness, the leptospires disseminate to visceral organs through blood stream of the infected persons^17^ and are excreted in urine from day 6 post-infection onward^18^. Therefore, the detection of leptospiral antigens using precise antibodies will be an alternative strategy for the diagnosis of leptospirosis during the early stages of infection.

Monoclonal antibody based antigen detection methods have been increasingly used in detection of many bacterial and viral infections^19^. Nguyen *et al*.,^20^ used MAbs for the detection of typhoid during the early phase of illness. Initially the MAb has been produced for whole cell lysate of leptospires which showed specificity to its serovar and failed to detect others^4, 21^. The monoclonal antibody 1H6 was produced against leptospiral lipopolysaccharide^22^. The 1H6 MAb based immunochromatography showed a sensitivity and specificity of 89 and 87% respectively. On the other hand, the recombinant proteins which are surface exposed and conserved have also been used to develop monoclonal antibodies with remarkable diagnostic efficiencies^23^. Leptospiral immunoglobulin-like (Lig) proteins are *in-vivo* expressed surface protein that has gained attention as diagnostic and vaccine candidate. Detection of leptospirosis using recombinant C-terminal LigA described achievable specificity values of 100% and 98% for IgG and IgM ELISAs, respectively^24^. Serodiagnosis of leptospirosis using chemically synthesised peptides of LigA showed higher sensitivity and specificity of 97.9% and 99.1% respectively^8^. Therefore, the chemically synthesised peptides of LigA can be considered as effective antigens for production of monoclonal antibody.

In the present study, we have computationally identified immunogenic B-cell epitopes of LK90 (LigA-90kDa), immunized mice with synthetic peptides and developed monoclonal antibodies. Two positive stable clones P1B1 (LK90_543_) and P4W2 (LK90_1110_) were selected and used in dot-blot ELISA to detect antigens. Peptide based monoclonal antibody offers high specificity in terms of monovalent antigenicity and *Leptospira* specific diagnosis. We have explored the applicability of the MAb based dot-blot ELISA for the detection of leptospiral antigen in urine samples. The dot-blot ELISA developed with these MAbs were found to be highly specific for conclusive diagnosis of acute leptospirosis during outbreak situations.

For specificity assays, the MAbs obtained were tested against several leptospiral serovars and other bacterial pathogens. The MAbs were able to detect the native proteins in all leptospiral serovars with a specificity of 100% and no cross reactivity with non-leptospiral bacteria as evidenced by Western blotting and IgG ELISA. A major downside of the present study is that antigenic preparations from other spirochetes including *Treponema/Borrelia* were not included. Considering the exertion and our expertise in the laboratory cultivation of *Treponema/Borrelia* the antigens has been discounted. On the other hand, the efficiency of our developed MAbs was validated by incorporating clinical cases confirmed for other spirochetal diseases (Syphilis and Lyme). Additionally, pondering the use of paired sera to demonstrate the seroconversion or four-fold rise in titre for the laboratory confirmation of leptospirosis, the cross-reactivity of our MAbs with other spirochetes was debarred. The present strategies of developing MAbs have opened new avenues for the development of MAbs for other spirochetal antigens.

Further the dot-blot ELISA was applied to experimentally infected- and noninfected-mice urine samples. The culture method was used as a gold standard comparison for the calculation of sensitivity and specificity^25^. The sensitivity and specificity of the MAb based dot-blot ELISA were found to be 100%. Such increased sensitivity could possibly be due to high concentrations of leptospires in the infected mice urine samples. According to Monahan *et al*.,^9^, infected rats could excrete high concentrations of *Leptospira* (10^7^ cells/ml) after 3 weeks of infection.

Urine samples from 32 patients with laboratory confirmed leptospirosis, 28 healthy humans, and 33 field rats were tested using the MAb based dot-blot ELISA. MAT was performed and used as gold standard for diagnosis of leptospirosis. The specificity was between 93 and 96% and sensitivity between 77 and 89%. The decrease in sensitivity might be due to nonspecific reactions with unknown substances in the urine. Therefore, pre-treatment of urine samples will be necessary to eliminate these substances. But there were no sensitivity issues when experimentally infected mice urine samples were used for dot-blot ELISA. This could possibly be due to the higher concentrations of leptopsires over the non-specific substances. However, further study is necessary in order to create simpler and more-cost-effective methods of pre-treating urine samples and to determine the range of leptospiral antigen concentration that could be detected without compromising the assay sensitivity.

The concentration of *Leptospira* that is usually found in urine from dogs ranges from 10^1^ to 10^6^ cells/ml^26^. The concentration of leptospires varies among animals with variable assay sensitivity. This is a limitation of our study and further improvements are needed for the wide applicability of the produced MAbs.

In conclusion, the MAb based antigen detecting dot-blot ELISA from urine sample has been developed for the diagnosis of acute leptospirosis. Since the test doesn’t need any special equipment including PCR, western blot apparatus, dark field microscopy maintaining of live leptospires and invasive procedure for the collection of specimen from patients, it is considered as an effective alternative strategy for diagnosis of acute leptospirosis in primary health care centres. And moreover dot-blot ELISA is simple, rapid can be performed in less than 8 hours (including incubation with MAb for 1 h, followed by washing for 30 mins, incubation of secondary antibody for 2 h, washing for 30 mins, and development, visualization and interpretation in 30 mins), inexpensive and affordable in developing countries and area where laboratory facilities are limited. Conclusively, the MAbs obtained were able to detect the native proteins in several leptospiral serovars as evidenced by Western blotting and IgG ELISA. The dot-blot ELISA developed with these MAbs is highly specific for conclusive diagnosis of acute leptospirosis during outbreak situations.

## Electronic supplementary material

Supplementary Information

## References

[1] Bharti AR (2003). Leptospirosis: a zoonotic disease of global importance. Lancet Infect. Dis..

[2] Douglin CP, Jordan C, Rock R, Hurley A, Levett PN (1997). Risk factors for severe leptospirosis in the parish of St. Andrew, Barbados. Emerg. Infect. Dis..

[3] Dupont H (1997). Leptospirosis: prognostic factors associated with mortality. Clin. Infect. Dis..

[4] Saengjaruk P (2002). Diagnosis of human leptospirosis by monoclonal antibody-based antigen detection in urine. J. Clin. Microbiol..

[5] Cumberland P, Everard CO, Levett PN (1999). Assessment of the efficacy of an IgM-elisa and microscopic agglutination test (MAT) in the diagnosis of acute leptospirosis. Am. J. Trop. Med. Hyg..

[6] Choy HA (2007). Physiological osmotic induction of Leptospira interrogans adhesion: LigA and LigB bind extracellular matrix proteins and fibrinogen. Infect. Immun..

[7] Palaniappan RU (2006). Immunoprotection of recombinant leptospiral immunoglobulin-like protein A against Leptospira interrogans serovar Pomona infection. Infect. Immun..

[8] Kanagavel M, Shanmughapriya S, Anbarasu K, Natarajaseenivasan K (2014). B-cell-specific peptides of leptospira interrogans LigA for diagnosis of patients with acute leptospirosis. Clin. Vaccine Immunol..

[9] Monahan AM, Callanan JJ, Nally JE (2008). Proteomic analysis of Leptospira interrogans shed in urine of chronically infected hosts. Infect. Immun..

[10] Koizumi N (2012). A new loop-mediated isothermal amplification method for rapid, simple, and sensitive detection of Leptospira spp. in urine. J. Clin. Microbiol..

[11] Faine S (1981). Leptospirosis–here, now. Pathology.

[12] Kanagavel M (2016). Multilocus sequence typing (MLST) of leptospiral strains isolated from two geographic locations of Tamil Nadu, India. Infect. Genet. Evol..

[13] Raja V (2015). *In Vivo*-Expressed Proteins of Virulent Leptospira interrogans Serovar Autumnalis N2 Elicit Strong IgM Responses of Value in Conclusive Diagnosis. Clin. Vaccine. Immunol..

[14] Natarajaseenivasan K, Boopalan M, Selvanayaki K, Suresh SR, Ratnam S (2002). Leptospirosis among rice mill workers of Salem, South India. Jpn. J. Infect. Dis..

[15] El-Manzalawy Y, Dobbs D, Honavar V (2008). Predicting linear B-cell epitopes using string kernels. J. Mol. Recognit..

[16] Raja V, Natarajaseenivasan K (2015). Pathogenic, diagnostic and vaccine potential of leptospiral outer membrane proteins (OMPs). Crit. Rev. Microbiol..

[17] Thiermann AB (1984). Isolation of leptospires in diagnosis of leptospirosis. Mod. Vet. Pr..

[18] Nally JE (2011). Comparative proteomic analysis of differentially expressed proteins in the urine of reservoir hosts of leptospirosis. PLoS One.

[19] Berry JD (2005). Rational monoclonal antibody development to emerging pathogens, biothreat agents and agents of foreign animal disease: The antigen scale. Vet. J..

[20] Nguyen NQ (1997). Diagnosis of enteric fever caused by Salmonella spp. in Vietnam by a monoclonal antibody-based dot-blot ELISA. Asian Pac. J. Allergy Immunol..

[21] Yan KT (1998). Development of an immunomagnetic antigen capture system for detecting leptospires in bovine urine. Res. Vet. Sci..

[22] Widiyanti D (2013). Development of immunochromatography-based methods for detection of leptospiral lipopolysaccharide antigen in urine. Clin. Vaccine Immunol..

[23] Coutinho ML (2007). Evaluation of the anti-LipL32 monoclonal antibodies potential for use in leptospirosis immunodiagnostic tests. J. Immunoassay Immunochem..

[24] Srimanote P (2008). Recombinant ligA for leptospirosis diagnosis and ligA among the Leptospira spp. clinical isolates. J. Microbiol. Methods.

[25] World Health Organization (WHO) (ed.). Human leptospirosis: guidance for diagnosis, surveillance and control. WHO Library, ISBN 924154589 **5**, 1–107 (2003).

[26] Rojas P (2010). Detection and quantification of leptospires in urine of dogs: a maintenance host for the zoonotic disease leptospirosis. Eur. J. Clin. Microbiol. Infect. Dis..

