# Supplementary material for: Peptide specific monoclonal antibodies of Leptospiral LigA for acute diagnosis of leptospirosis
Source: Sci Rep. 2017 Jun 12;7:3250. doi: 10.1038/s41598-017-03658-0 (PMC5468321; doi:10.1038/s41598-017-03658-0)
Supplement: Supplementary file 1 — Supplementary Information [file 41598_2017_3658_MOESM1_ESM.pdf]

Supplementary Information

Peptide specific monoclonal antibodies of Leptospiral LigA for acute diagnosis of leptospirosis

Murugesan Kanagavel[1], Santhanam Shanmughapriya[1#], Kayanam Vijaya Lalitha Aishwarya[1], Karuppiah Ponmurugan[2], Kasi Murugan[2$], Naif Abdullah Al-Dhabi[2], Kalimuthusamy Natarajaseenivasan[1]*

[1]Medical Microbiology Laboratory, Department of Microbiology, Centre of Excellence in Life Sciences, Bharathidasan University, Tiruchirappalli, TN 620024, India.

[2]Department of Botany & Microbiology, College of Science, King Saud University, Riyadh 11451, Saudi Arabia.

Present Address: [#]Center for Translational Medicine, School of Medicine, Temple University, Philadelphia, PA 19140, USA. [$]Department of Biotechnology, Manonmaniam Sundaranar University, Tirunelveli, TN 627012, India.

Table S1: Details of the day of illness for samples included in the study

Serogroups	No. of positive Sera	Mean number of days after onset of symptoms
Autumnalis	20	8.6
Australis	5	9.6
Canicola	2	9.5
Grippotyphosa	1	8
Ballum	1	7
Pomona	1	9
Javanica	2	7.5

Table S2: Age and sex wise distribution of the confirmed cases of leptospirosis included in the study

Age Group	Sex		No. and % of cases (N = 32)
	Male	Female	
0-9	0	0	0 (0)
10-19	4	0	4 (12.5)
20-29	3	2	5 (15.6)
30-39	6	3	9 (28.1)
40-49	8	4	12 (37.5)
50-59	2	0	2 (6.3)
60-69	0	0	0

Table S3: Median MAT titers of 32 sera from laboratory confirmed cases of leptospirosis and 33 rodents

Serovars	Human		Rodent	
	Number of samples (%) N = 32	MAT titre	Number of Samples (%) N = 33	MAT titre
Autumnalis	20 (62.5)	1:320	13 (39.4)	1:160
Australis	5 (15.6)	1:160	2 (6.1)	1:80
Canicola	2 (6.3)	1:160	0 (0)	
Grippotyphosa	1 (3.1)	1:80	2 (6.1)	1:80
Ballum	1 (3.1)	1:80	0 (0)	
Pomona	1 (3.1)	1:80	2 (6.1)	1:160
Javanica	2 (6.3)	1:80	14 (42.4)	1:160

Table S4: Prediction of B-cell epitopes by BCPred and antigenicity determined by VaxiJen score

Protein	Epitopes	BCPred score	VaxiJen Score
**LK90** (0.5405)	T_{610}DNSNSDITNQVTWNSSNTD$_{629}$	0.999	0.5360
	S_{543}NAQKNQGNAYGAATGATDI$_{562}$	0.993	1.1390
	V_{1167}TWSSSNPTVVSISNVDDER$_{1187}$	0.985	0.5326
	D_{1110}HHTQSSYTPVTVTESGIVN$_{1129}$	0.984	0.6536
	S_{799}AVTWHSSNNSVATISNTKG$_{818}$	0.984	0.5619
	F_{880}TDNSKKDITDQVTWNSSS$_{898}$	0.975	0.4047
	K_{459}GLGKAHAVGDTTITATLGK$_{478}$	0.975	0.7303
	S_{1078}SNPSSVVIENTPGKKGLAF$_{1096}$	0.961	0.7041

Table S5: Evaluating the cost-effectiveness of the Dot-Blot ELISA

Parameters	Values	ICER (C1-C0/E1-E0)
Cost of dot-blot ELISA (C1)	41.47	$ 8.7/QALY
Cost of dot-blot ELISA (C0)	93.625	
Effect of dot-blot ELISA (E1)	11[a]	
Effect of dot-blot ELISA (E0)	5 [b]	

[a-] QALY for Dot-Blot ELISA: Quality adjusted life, patients with no-fever days in 15 days' limit analysis. [b]QALY for MAT: Quality adjusted life, patients with no-fever days in 15 days' limit analysis.

Figure S1: Specificity of MAbs P1B1 and P4W2 for detection of Leptospira

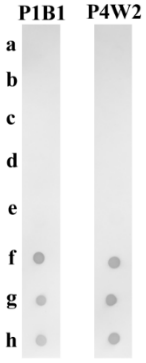

One microgram of heat extracted proteins from clinically isolated bacterial strains and MACS leptospiral strains were dotted and probed with indicated MAbs; Heat extracted proteins from *E.coli (a), Staphylococcus aureus (b), Salmonella typhi (c), Serratia*

marcescens (d), Pseudomonas aeruginosa (e), *L. interrogans* serovars Autumnalis strain Akiyami *(f)*, Bangkinang *(g)* and N2 *(h)*.

Figure S2: Determination of antigen concentration for MAb based Dot-blot ELISA assay

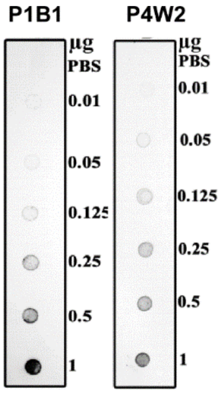

Various concentrations of heat extracted antigen from *L. interrogans* serovar Autumnalis strain N2-MACs was probed with the produced MAbs.

Figure S3: Optimizing MAbs concentration for use in antigen capture by Dot-blot ELISA

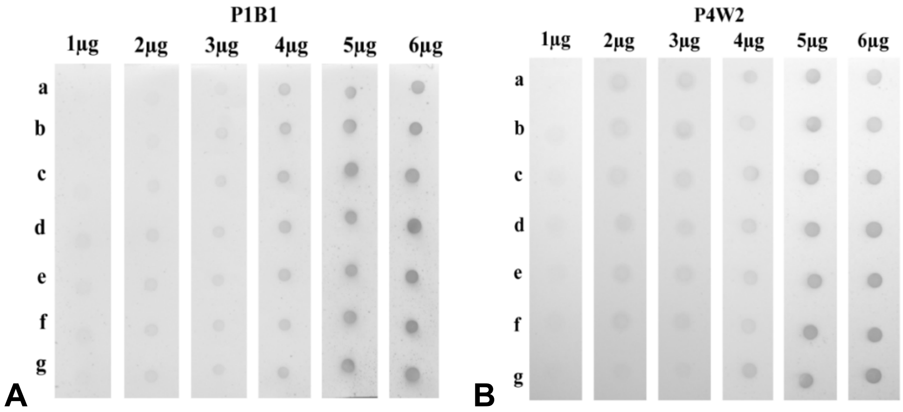

Optimizing the concentration for P1B1 (**A**) and P4W2 (**B**) MAb. (a to g): 1 µg of protein extracted from *L. interrogans* serovars Autumnalis strain Akiyami, Bangkinang and N2, serovar Australis, serovar Icterohaemorrhagiae, serovar Pomona, serovar Bataviae

were probed with various concentrations of MAbs from 1 to 6 µg per ml by Dot-blot ELISA.

Figure S4: Evaluation of MAbs in dot-blot ELISA using experimentally infected mice urine samples

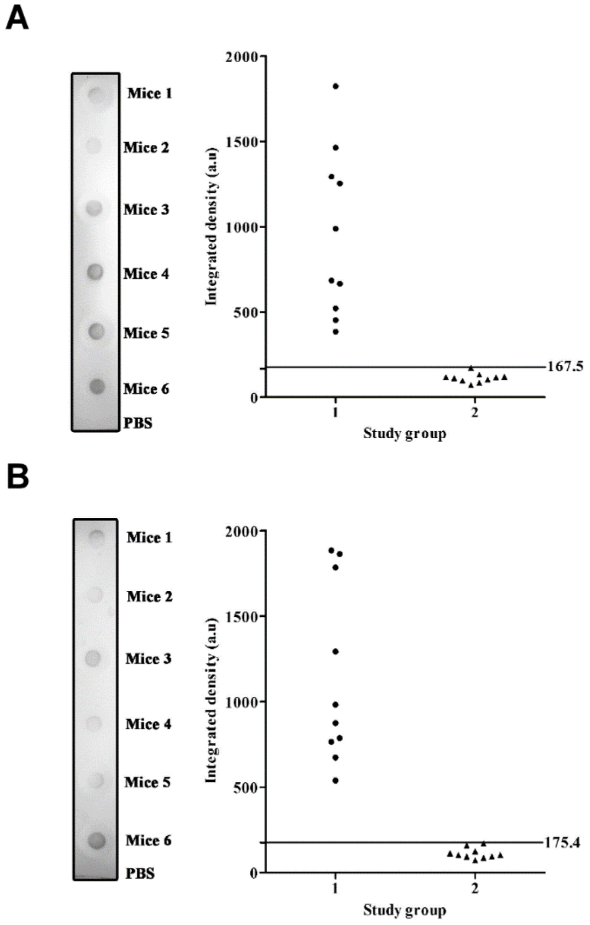

Study groups are indicated on the *x* axis and intensity of the dots on the *y* axis. Sensitivity and specificity of the MAb based dot blot ELISA for P1B1 (**A**) and P4W2 (**B**) to detect leptospiral antigens in mice urine samples are shown. Study groups: Group 1 – experimentally infected mice urine samples; Group 2- healthy controls. The dashed line represents the cut-off values with the absolute cut-off values on the right.

Figure S5: Evaluating the cost-effectiveness of the Dot-Blot ELISA

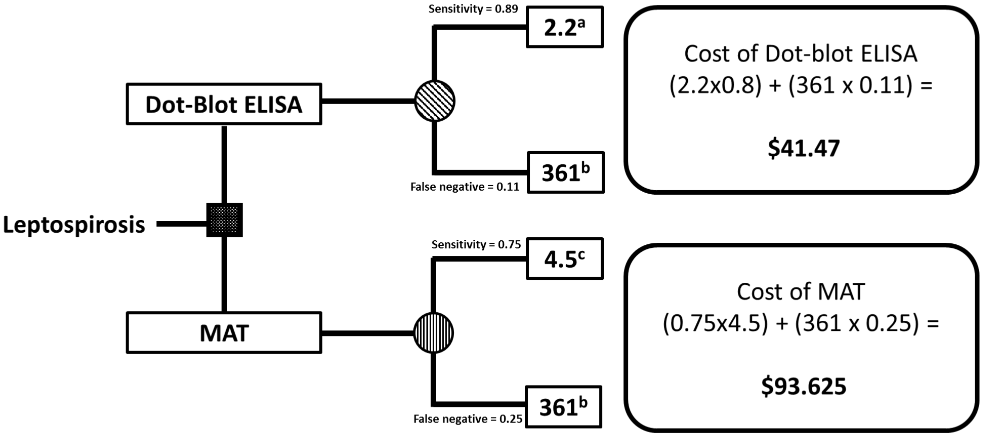

[b]- approximate cost for Dot-Blot ELISA

[c]- approximate estimate of hospital costs, including intravenous antibiotics, other treatment.

[d]- approximate cost for MAT

Calculated costs for two strategies (tests) = (cost of consequence 1 x probability 1 (sensitivity)) + (cost of consequence 2 x probability 2 (1 -sensitivity))
